# Feminist Approaches to Gender Equity in Perú: The Roles of Conflict, Militancy, and Pluralism in Feminist Activism

**DOI:** 10.3389/fpsyg.2022.834763

**Published:** 2022-03-18

**Authors:** Shelly Grabe

**Affiliations:** Department of Psychology, University of California, Santa Cruz, Santa Cruz, CA, United States

**Keywords:** conflict, militancy, pluralism, feminism, Perú, gender equity

## Abstract

For the past several decades, coordinated efforts from within the women’s social movement in Perú have led to groundbreaking legislation surrounding gender equity – for example, the National Gender Equality Policy of 2019 and the Gender Parity Law of 2020. These institutionalized policy changes mark milestones on the path to gender equity, certainly in Perú, but activist efforts that targeted these outcomes can inform women globally. The current study investigated key components of feminist activism by social movement actors themselves through the use of testimonio with nine key leaders in the movement. Using a liberation psychology approach and thematic narrative analysis, the findings suggested three key components of feminist activism: conflict, militant identity, and pluralism that were critical in processes of change. Centering majority world women’s voices contributes to the production of knowledge regarding approaches to gender equity, in particular because much that has been written about feminist action in psychology has been produced among samples of white women in the United States. Implications for understanding how the findings have the potential for global change are discussed.

## Introduction

Gender inequities prevail in most countries throughout the world, despite ongoing attempts to eliminate them. Across the globe, prospects for change are limited by social norms, practices, and policies that are rooted in gender discrimination and bias. Throughout Latin America, feminist activists have responded by working toward democratization in an effort to address gender equity (e.g., Sternbach et al., 1992; Tobar et al., 2009). The women’s social movement in Perú, specifically, has a targeted focus on recognition and redistribution of political power as central to processes involving equity. The Peruvian women’s movement emerged like many other Latin American social movements, in the 1970s and 1980s in the context of military-backed rule and armed conflict (Barrig, 1994). By 1991 historian, scholar, and feminist activist Virginia Vargas described the women’s movement in Perú as “one of the largest on the continent, possibly having the most diversified expression and organizations” (Vargas, 1991, p. 7). Related to the collective organizing efforts among women in the movement, in 1996 Perú established a women’s ministry as the governing body on national policies for women (Ministry of Women and Vulnerable Populations; MIMP).

In more recent years, coordinated efforts within the women’s social movement have led to ground-breaking advances administered by the MIMP. For example, in April 2019, the MIMP published Perú’s National Gender Equality Policy (Decree 008) with the strategic goals to eliminate structural discrimination in areas related to the determinants of gender inequity. For example, there are specific goals aimed at reducing violence against women, guaranteeing sexual and reproductive health rights, and reducing institutional barriers existing in the public and private spheres between women and men. The effect of the Peruvian National Gender Equality Policy has already been witnessed. In June 2020, the Peruvian Congress passed a ground-breaking Gender Parity Law (Law No. 31030) requiring that 40% of all congressional candidates be women beginning in 2021, 45% be women by 2026, and 50% be women by 2031. These achievements mark milestones on the path to gender equity and are a defining moment, certainly in Perú, but activist efforts within a movement that targeted these outcomes can inform women globally.

Therefore, women’s social activism in Perú represents an important case from which to translate and adapt knowledge about feminist processes aimed at gender equity. Moreover, given that much of what we know about feminism is derived from a white, U.S.-based standpoint (e.g., Moon and Holling, 2020), it is imperative that we include more perspectives of majority world^1^ women in our understanding of feminist activism. The aim of the current study was to investigate key components of feminist activism by social movement actors in Perú, as articulated by the women themselves.

### Social and Historical Context of the Women’s Movement in Perú

The election of a civilian president in 1980 opened space for alternative forms of political engagement following decades of military rule and armed conflict in Perú. For example, new leftist political parties emerged in Perú driven by a focus on democracy and an economic crisis. However, like many leftist-leaning movements at the time, as social class issues became a political focal point, women’s issues were side-lined to the interests of male-dominated party politics. For example, although many women played a very active role in the politics that emerged in the late 1970s in Perú, they never gained leadership positions and were therefore not able to get involved as a means to further their own rights (Huls, 2011). As a result, during the late 1970s, many women decided to separate from the leftist political parties to form feminist organizations that would address gender inequity as a central priority independent from political party affiliation – using the opportunity to democratize their participation and increase demands on the government to view them as equals (Huls, 2011). The newly formed organizations that centered on the advancement of women formed the backbone of the current women’s social movement in Perú. Two of the most important women’s organizations within the feminist movement date back to 1979 and 1980, respectively, the Flora Tristán Center for Peruvian Women^2^ and the Manuela Ramos Movement; both organizations founded by women who defected from party politics to focus on feminist activism as a means to enact social change.

The conflict between leftist political parties and women’s rights groups grew as the women’s movement ballooned in the 1980s amidst economic crisis and extreme poverty (Vargas, 1991). One way this manifested is through how activist women identified themselves. For example, at the time *militantes* (militants) referred to individuals who were left-wing political activists, whereas *feministas* (feminists) referred to women who operated in various collectives focused on gender justice (Sternbach et al., 1992). And while there was an obvious overlap between the two, and many feminists found political space within the larger opposition struggle launched by the left, it was also true that the practices of the male-dominated progressive leftist party invariably relegated a back-seat status to women’s issues. Therefore, *feministas* were those who advocated for political autonomy and held the position that neither capitalism nor socialism could eliminate women’s oppression in the absence of addressing patriarchy. In other words, adequately addressing women’s demands would require social action outside of the existing political parties that centered different platforms for action. On the other hand, women who identified as *militantes* believed addressing the androcentrism of the leftist parties was not insurmountable. This resulted in a tension between politically active Peruvian women who identified first and foremost as party *militantes* and those who identified as *feministas*– the differences largely surrounded strategies that should be adopted to end women’s oppression. There were also women who proposed a *doble militancia* perspective, whereby concurrent participation in, and dual commitment to, political party and feminism could occur hand-in-hand, though this was difficult to impossible to achieve in practice.

In addition to the conflict presented as a result of the male dominance in political participation, the seeds of the women’s movement also introduced a conflict between women themselves to the extent that the initial organizations serving as the foundation of the women’s movement did not fully capture demands that were representative of all women in Perú. Specifically, the efforts of a group of middle-class women who defected from party politics in the 1970s did not adequately recognize the general dissatisfaction of women from other social locations or backgrounds. An early result was that three “streams” of activism emerged in the movement reflecting the heterogeneity of the women involved, which Vargas describes as a plurality of processes reflecting an outstanding feature of the Peruvian women’s movement (1991). The first “stream,” labeled “feminist,” focused on denouncing a sex-gender classification system that was responsible for subordinating women. The second “stream,” viewed as the “popular women’s” efforts, sought practical solutions to the demands that sprung from traditional gender roles. And the third “stream,” which focused in traditional political spaces, was aimed at demanding more female participation at decision-making levels. Although women from the three “streams” were often in solidarity, the processes “mixed different realities, experiences, influences, and take on specific forms of expression in their interrelation with the contradictions underlying women’s lives, such as class, race, age, geographic location, etc.” (Vargas, 1991, p. 10). The streams, because they shed light on the different experiences of women, demonstrated a way of prioritizing difference and transforming it into a driving force of collective actors. Although these distinct streams are not observed today, the streams served as the backbone of a movement that thrives on approaches that are inclusive of diverse realities.

The feminist activism in Perú in the early days can be further contextualized by the emergence of a focus on women’s human rights that was initiated with the United Nations Women’s Decade that began in 1975. Throughout that decade, gendered projects expanded throughout all of Latin America with activists undertaking a specifically feminist vision of politics, culture, and society. One example was the regional Latin American and Caribbean Feminist *Encuentros* – or conferences – which began in 1981 and were held every few years to develop meeting spaces for feminist activists to strategize ways of eliminating various forms of marginalization and oppression experienced by women. The second regional *Encuentro* was held in Lima, Perú, in 1983. This meeting set the stage for a “theoretical understanding of Latin American patriarchy in all of its material, ideological, cultural, linguistic, institutional, and sexual expressions, deepening and advancing the movement’s analysis of gender power relations and how these intersected with other relations of power in Latin American societies” (Sternbach et al., 1992, p. 412). These meetings, and the strategies that emerged from them, reflected a core value Peruvian women placed on approaches that were inclusive of the diversity of women’s experiences.

In sum, early confrontations and conflicts appear to have shaped the women’s movement and contributed to how feminism in Perú is practiced today. Moreover, an exchange of ideas that recognizes plurality of experience, though initially rooted in conflict, seemed necessary for the movement to grow and to inform strategies that would be able to address institutionalized gender inequity. In recent years the feminist activism in Perú has been ground-breaking, though there is little empirical evidence that details what led them to this moment. Because the Peruvian women’s movement reflects a rich location from which to analyze the potential for change, the current study will investigate key feminist principles that characterized a movement that led them to where they are today.

### Psychology of Liberation

Scholars have conceptualized different processes to recognize and theorize how people move toward equity and justice in their worlds. For example, a predominant concept within psychology used to describe this process has been empowerment. Julian Rappaport–writing in community psychology in the 1980s– inspired numerous scholars to adopt this concept in elucidating social justice initiatives (Rappaport, 1987). Empowerment has also been a key theme in feminist development practice since the 1980s. However, as empowerment initiatives grew over time, they became buzzworthy “in the service of one-size-fits-all development models” used to strengthen international development initiatives’ moral authority and perceived efficacy while being de-coupled from the initial intention to challenge gender inequity and promote structural change (Cornwall, 2016). Over time, it became common for interventions to overlook structures and systems that maintain inequity in favor of assisting individuals portrayed as victims who were presumably unable to circumvent their mistreatment without the help of “benevolent” outsiders (Alexander, 2006; Prilleltensky, 2008). Moreover, the focus on economic dimensions of empowerment became a thorn for many critical scholars who view economic empowerment as related to neoliberal aims, whereby “empowerment” initiatives began to uphold current power imbalances by becoming a tool with which to produce self-governing and self-caring social actors (Hickel, 2014).

In the context of large systems of inequality, liberation psychology may be better-suited to examine how women work toward equity because it recognizes that “limit situations,” or circumstances that constrain people’s lives, are also places where possibilities begin (Martín-Baró, 1994; Montero, 2009). From a liberation psychology perspective, through awareness of and dialogue about limited situations, a critical analysis can lead to action. Specifically, as awareness of context-specific patterns that limit life circumstances develop (i.e., situations whereby gendered power differentials are a result of structural inequities rather than the fault of individual women), possibilities for action are explored and further awareness develops in a cyclical process (Moane, 2010). An overemphasis in traditional psychology on topics such as empowerment may preclude an understanding of how activism that evolves within limited situations can facilitate the transformation of social conditions associated with inequity.

In response to a growing host of injustices related to gender inequity worldwide, critical feminist psychologists have underscored the need for investigations that examine the ways women resist power-based constraints to demand conditions of gendered justice (Moane, 2003; Brodsky, 2009; Lykes and Moane, 2009; Grabe, 2012, 2015, 2016; Grabe et al., 2014). Considering an approach that focuses on women’s activism is compatible with Brazilian social theorist Freire’s (1970) articulation of liberation in which he suggests that individuals are most likely to change the inequitable circumstances in which they live by challenging social structures (Moane, 2003; Brodsky et al., 2012). According to Freire (1970) this process involves the concept of *conscientización* or a process in which those working to create bottom-up social change participate in an iterative, ideological process whereby analysis and action develop together in a limited situation. Among the first steps in this process is the development of critical understandings of how adverse social conditions (e.g., male dominance) undermine well-being (Prilleltensky, 2008). Critical psychologist Montero (1994) suggests that the understandings that emerge in limit-situations are used to *problematize* social conditions, in other words to view the conditions, rather than individuals, as problems that require solution. Problematizing social conditions results in *deideologizing*, or reconstructing understandings of lived experience based on rejecting dominant ideologies that justify social oppression (Montero, 1994, 2009).

In the past two decades, theoretical and analytical approaches from liberation psychology have been increasingly used to investigate how individuals develop their capacities to resist oppression and engage as decision-makers for transformative change (Moane, 2006; Montero, 2007; Grabe, 2015, 2016; Dutt and Grabe, 2019; Grabe and Dutt, 2020; Grabe et al., 2020). In 2009, Lykes and Moane produced a ground-breaking Special Issue by identifying researchers who were applying feminist liberation psychology to the work of feminist social movements by focusing on liberatory processes in their investigations in India, Guatemala, Pakistan, and Portugal (e.g., Crosby, 1999; Chaudhry and Bertram, 2009; de Oliveira et al., 2009; Madrigal and Tejeda, 2009; White and Rastogi, 2009). A small but growing literature since that time has begun to apply this theorization to the development of women’s liberation in the context of gendered inequity elsewhere (Afghanistan, Nicaragua; Brodsky et al., 2012; Dutt and Grabe, 2014; Grabe et al., 2014). Still others have examined women’s work within social movements by examining pathways to activism or how women become committed to social change (McGuire et al., 2010; Dutt and Grabe, 2014; Savaş and Stewart, 2019). The aim in the current study is to build on this literature by investigating the key components of activism among women in Perú who were working to transform their structural circumstances by exerting their right to politically participate as active decision-makers contributing to the creation and sustainability of an equitable society.

In centering majority of world women’s voices, the current study also takes a decolonial approach by using the standpoint of feminists in Perú to contribute to the production of knowledge about gender equity (Macleod et al., 2020). Much that has been previously written about feminist action in psychology has been produced among samples that are overwhelmingly white and located in the United States. The exploration of important factors involved in feminist activism in a majority world country fits into the global focus of this Special Issue by attempting to call into attention understandings of feminist action that may expand beyond what is present in the literature.

## Materials and Methods

This study was designed in consideration of Mohanty’s (2003) assertion that understanding women’s struggles for gender equity must involve illuminating “third world women’s” engagement with resistance to oppressive authorities and governments. To best understand how feminist activism was employed in efforts toward equity in Perú, the current study privileges the standpoint of the activists working directly on issues related to the advancement of women’s rights (Maddison and Shaw, 2007). The method used for interviewing activists in this study centered on the use of *testimonios* which allow for an explicitly political narrative, or life story, describing and resisting oppression (Chase, 2003). *Testimonios* have been widely used to interview Latin American activists among scholars documenting women’s activism (e.g., Randall, 1981, 1994; Menchú, 1984; Grabe, 2016). Although this method can showcase the often invisible and undocumented activity among women who are underrepresented in the construction of knowledge, it is also true that what is reported in this manuscript was filtered through the author’s social location, which involves the power and privilege afforded to a middle-class white woman working within a university in the United States.

### Participants and Procedure

This project began with a scholar-activist collaboration in partnership with the Global Feminisms Project (GFP; Lal et al., 2010). The GFP is a collaborative international project, housed at the University of Michigan, which has conducted, examined, and archived interviews with women involved in feminist activism, social movements, and women’s studies departments in 9 different countries prior to Perú.^3^ The GFP site publicly archives video and transcript forms of the interviews conducted with women and allows open access to the material for teaching and research purposes. To initiate inclusion of women from Perú to the project, the author established a collaboration with one of the directors (Diana Miloslavich Túpac) of the Flora Tristán Peruvian Women’s Center. Flora Tristán is a leading feminist institution that was created in 1979 when women broke from the leftist political parties to establish non-profit civil associations focused on women’s rights. Today, Flora Tristán exists within a multi-sector, coordinated mobilization of women in Perú with the mission to combat the structural causes that restrict women’s citizenship.

Because an underlying goal of activist research is a reconfiguration of knowledge production that shifts power and control into the hands of the marginalized (Borda, 1985; Sandoval, 2000), the women selected for the interviews were identified by the director at Flora Tristán. We interviewed nine women who reflect the multicultural and intersectional approach to gender equity that the movement actors place on difference. The interviewees have diverse backgrounds that reflect the complex identities included in the feminist movement. For example, the sample included Indigenous leaders, scholars, congresswomen, directors of key feminist organizations, obstetricians, *trans* activists, and youth leaders. The women were from several different regions of the country and ranged in age from their early 30s to their 70s at the time of the interview.

All of the interviews occurred in Spanish via Zoom during the COVID pandemic in the Summer of 2020. The interviewer was a Lima-based activist, documentary director, and professor whose own work was focused on gender justice. All of the interviews were preceded by a conversation that explained how the woman’s *testimonio* would be part of the GFP and each interviewee was granted permission for their names and interviews to be publicized. The interviews lasted approximately an hour and the videos and transcripts are reproduced in full (i.e., unedited) on the Global Feminisms website. Questions in the interview followed an oral history format that was largely consistent across the GFP sites. All of the interviews were transcribed verbatim by a team of undergraduate students and translated into English by hired staff at the Global Feminisms Project.^4^ Analysis of the interviews was conducted on the English transcriptions.

### Analytic Strategy

Thematic narrative analysis was used to examine the testimonios and identify feminist principles that characterize the activism employed within the movement to address gender inequity. In particular, narrative analysis was used to examine the meaning the women attached to their active engagement in women’s rights activism (Marecek et al., 1997). The analyses of the interviews focused on identifying recurring themes that illustrated key feminist principles that women in Perú employed toward change. In interpreting the findings, attention was given to the ways in which the processes operated across women’s life stories within a coordinated movement (Lieblich et al., 1998; Riessman, 2000). The author and a graduate student read, re-read, and met regularly to discuss the interviews in their entirety, noting overarching patterns and ultimately focusing on a thematic content analysis of the interviews to identify patterns of similarity across the women’s stories that occurred repeatedly within the interviews (Lieblich et al., 1998; Braun and Clarke, 2006).

## Findings

Three themes emerged in the results that illustrate key feminist principles that women in Perú employed toward change: (1) awareness of context-specific patterns of **conflict** was used to progress goals, rather than being viewed as situations that presented obstacles, (2) self-labeling as a “**militant**” emerged as an important way to declare an ideological and active commitment to engaging *feminism* as a legitimate platform for action and, (3) feminist activists approached liberation through the practice of **pluralism**, or an approach that was inclusive of the diversity of women’s realities and experiences throughout the country, rather than using a homogenous process or one that celebrated individual efforts and ideas.

**Theme 1: Conflict: Viewing difference/disagreement as a positive experience to take the movement further.**

Not surprisingly, given what is known about the historical context of the women’s movement in Perú, many women in the sample articulated experiences of direct conflict. However, the examples shared were not described simply as hardship but rather reflected how activist women viewed adverse conditions as opportunities that warranted action. For example, Lourdes Huanca Atencio, who was the founder and president of an Indigenous women’s organization,^5^ described experiences riddled with conflict as she gained experience focusing on women’s rights:

It became my main life commitment when I took on the leadership of the Peasant Confederation of Perú (FENMUCARINAP). A mixed, macho, patriarchal organization is the idiosyncrasy of the agrarian sector, the peasant sector of the Indigenous peoples. It has helped me a lot to have a firmness of wanting to advance and learn because being in a space as a national leader, you had a space to learn. Of course, it hasn’t been easy because within that space of mixed organization, they tell you “hey, you’re a woman. What are you going to operate? What do you know? Go cook, go warm up, go get us some coffee.” I sometimes felt discriminated against. but I think that we must also value, with great strength, the strength of our feminist comrades….I would go to my partner Flora Tristán with Diana Miloslavich^6^ and DEMUS^7^ with María Ysabel Cedano…. they were the ones who gave me strength and fortitude. I have resisted five years in the CCP,^8^ with strength, courage, decision and position. Many times the difference was that the advisors were taking this agreement for the leaders and I was the leader of the National Board of Directors but however, as I was a woman, there was no respect. When I left, I was empowered by the Floras, by the feminists, and I returned more empowered to the CCP. In other words, Flora was my training as a woman of resistance. With the DEMUS sisters, with all of them. They taught me about resistance. I returned to the CCP with strength. …I remember in an assembly that only the men were there, and they wouldn’t let me in, so I pushed them away and I said to the advisor, I rolled up my sleeves like this, “Are you going to let me participate here or not?” Then the comrades were cold, they didn’t know whether to respond, not to respond because I remember that I spoke to them “you have to lead and you are not going to kick me out because I am a national leader as well. And if you do, I’m going to sue you and I’m going to hold a press conference.” I was armed like that because that’s also how I was trained, so that’s been useful for making decisions. The next time they let me in. The issue was also how to fight with them in order to get the space to make decisions.

In describing the conflict she experienced in attempting to gain a leadership position within an organization, Lourdes recognizes that the initial conflict came in the form of discrimination with a negative impact on her well-being. However, her statement that “as a woman, there was no respect,” reflects awareness of a dominant ideology that justified discriminatory treatment toward women in a manner that functioned to uphold inequity. Deideologizing, or resisting this perspective, led her to problematize the condition – rather than herself – and seek solidarity. Through solidarity, she credits feminist processes for training her to be a “woman of resistance.” An ideological process, whereby her analysis of the situation and resulting action, led Lourdes to emerge from adverse social conditions as a decision-maker – one who demands gender justice by focusing on actualizing women’s rights and leadership within her organization. At the time of the interview, Lourdes was serving as the president of FENMUCARINAP.

In another example of viewing conflict as opportunity, Gahela Tseneg, who was only 27 when interviewed, spoke about the conflict introduced between men and women as the concept of political parity^9^ began to gain traction in Perú. Although not seated, Gahela had run to become the first transgender woman in Congress in the January 2020 elections, campaigning with what has been described as one of the most courageous and intersectional programs of the emerging and diverse Peruvian left. When speaking about political parity, she shared this:

One of the colleagues from Puno stood up and said to me, “colleague Gahela, but why parity?” I said, parity guarantees the participation of women, but it doesn’t guarantee the participation of women in diversity, that will depend on the political organizations, it will depend on the advocacy that we do. I felt challenged, but at the same time, I also feel that these processes of reflection are necessary. That is to say, we can’t just build from the desk, we have to build there in practice, when women are going through these types of situations. Where they are facing disputes within the communities, where the places where they take office, and the men stop going to the assemblies just to sabotage their efforts. It seems to me valid to reflect on this. It is something that forces us to have a much more reflexive process of what this implies and how we implement mechanisms that guarantee the participation of women in all their diversity, or how we guarantee the participation of young women, LGTBIs, in these spaces.

Gahela’s *testimonio* reflects how critical it is for progress that analysis and action develop together when she pointedly explains that conflict forces activists to be reflexive when considering how to implement mechanisms that will have a direct impact on the participation of women. The examples of conflict that she shares (i.e., disputes, sabotages) are contextualized as social problems that need a solution, rather than as issues that individual women ought to confront. Gahela’s *testimonio* provides an excellent example of the importance of awareness and reflection in processes involving liberation meant to implement successful mechanisms for change.

Tarcila Rivera Zea, a Quechua activist who has dedicated nearly 40 years of her life to defending and seeking recognition for the Indigenous people of Perú, also spoke candidly about the role of conflict in building resistance to inequity. She is a leader of CHIRAPAQ (Centro de Culturas Indígenas del Perú; Center for Indigenous Cultures of Perú), an Indigenous association that, for 30 years, has promoted the affirmation of identity and the recognition of Indigenous rights in the exercise of citizenship, with a special commitment to Indigenous children, youth, and women. Tarcila shared how conflict was at the root of founding this organization:

Knowing that there are conflicts -- and there is an imbalance in our relationships - we decided to open Chirapaq in a context of political violence in Perú in ‘86. I’m from a community in Ayacucho. and so I looked at the community of origin, the context of the big family and everything that was happening in Ayacucho in this tremendous violence between the State and the Shining Path,^10^ in which the more traditional communities were alien to that struggle, for example, my community was totally alien to that struggle and they didn’t know what was going on and they died, like cannon fodder. …All of this meant that people who had never left the community had to go out and defend their lives. This context had a great impact on us, and we got together as a small group and founded Chirapaq, first thinking about how the creators and producers of culture saw their lives and cultural continuity threatened in this conflict in a war that was not ours. Then we emerged strategically.

             …

We, as Indigenous women and as Chirapaq itself had conflicts within the movement and from outside. Within the movement, we were seen as an NGO,^11^ and the NGOs, at that time, were very questionable because the traditional leaders said that working with projects and with money in this form of association, was to use the people and to use the issue economically. So it was very difficult for us, it was very difficult for me to resist [laughter] to explain and to make people understand that we, too, as women, have a role in building the movement.

Tarcila’s testimony details how power imbalances are at the root of conflict. She describes “tremendous violence,” which is perhaps the epitome of conflict, as the foundational context that led to the formation of an organization devoted to Indigenous rights with a focus on women. Nevertheless, founding the organization did not lead to an end in conflict, but rather introduced conflict of a different sort. The new conflict that emerged was rooted in distrust among Indigenous communities that feared the organization would operate as an NGO whose funding would be driven by an outside agenda that did not have Indigenous rights at the forefront of their priorities. Tarcila’s account reflects that the central efforts to survival for her, in her role as a female leader within an Indigenous organization, were all characterized by conflict. An iterative process that continued to focus on structural power differences is what allowed her to survive.

Another Indigenous activist and feminist in Perú, Tania Pariona Tarqui, responsible for the Women’s Program in Chirapaq at the time of the interview, shared experiences of the current conflict. Tania is a young Quechua leader (she was 36 years old at the time of the interview), feminist, politician, human rights activist and former Congressperson. When asked about her experience as a woman working for organizations, she shared these reflections:

There are barriers that are institutionalized, that are part of the normalized structures. …You ask yourself why this happens? Why has it been normalized? All of a sudden you are living discriminations that you are resolving on your own, that you face alone. And if you have an organization to back you up, and you know that what is happening is wrong, you can take action to change that reality. The organizational space has been a support for me to not allow these violations, especially discrimination, to end up diminishing capacities…. When you have a public function and have a position of power or authority, it is up to you to ensure that these practices are punished, reported, and made aware of, and that you have to be the first to report what you are experiencing. On the one hand, it’s something much more complex because you have a commitment not to yourself but to others. So it helps you to have that strength, to always carry the challenge of not allowing that to be a standard practice….In my organizational process, we denounced cases of discrimination, we started campaigns against racism and discrimination, together with our Afro-descendant brothers and sisters. We made another international denunciation before the United Nations. We were part of the process of reflection, and of debate…. And for me to exercise in the parliament a function like the one we had, was possible thanks to the social support, collective, the organizations, the collectives of women, the Indigenous peoples, all of them that obviously motivate you to not lower your guard, nor to feel that you are alone, right?

  .

  In Perú I think we are in a process of strengthening and affirming our identity, which is becoming clearer and firmer. And perhaps because of the hard context that touches the lives of both Andean and Amazonian peoples, the denial, indivisibility, repression, territorial dispossession, the extractive presence, where there is always social and environmental responsibility, is making the Indigenous leadership emerge with force. **What becomes oppression and a problem, for us also becomes an opportunity.**

In addition to describing discrimination, as did Lourdes, the conflicts experienced by Tania led her to recognize that structural constraints interrupt opportunities for equity because they are institutionalized. The conflict described by Tania is addressed by problematizing how women are treated and recognizing the solution lies in solidarity that challenges the institution of patriarchy. Taking this approach allows protection from the potential for “diminished capacities” that would further strengthen inequity. As a former member of congress, Tania viewed it as her responsibility to use the service role to challenge the structures that disadvantage women collectively and to use a position of power or authority to denounce the mistreatment of women as standard practice. She saw the conflict introduced by discrimination as one that fuels campaigns and international solidarity. Moreover, she attributes the force of Indigenous leadership (of which she is among) to the harsh context of conflict when she shares her philosophy that from oppression rises opportunity.

Finally, Virginia Vargas, 75 years old at the time of the interview, was a founding member of the Flora Tristán Center, an internationally recognized activist and scholar with over 40 publications, and a recipient of a UNIFEM Award at the United Nations’ 4th World Conference on Women in Beijing (1995). She also described the important role of conflict in the building of the women’s movement:

I define myself more as an activist that reflects about our practice. Because I reflect about our view, our politics, new tendencies, the new conflicts. With conflicts is how a movement grows, or else it would really be more or less static, which is something that feminism is not. I believe that we were compromising with the construction of this movement and we fought for autonomy not only in regard to this organization but also of ideas…One of the leaders in the initial lesbian movement. comes and tells me “I want to talk to you, because you always support, you always say you are with us, you go to the mobilizations but let me see where is that here in the journal *Viva*?” Everything was heterosexual of course, there was no lesbianism, then well, I want to bring back the conflict as a substantial part to our advancement.

Vargas introduces an example of conflict that was not shared in the other *testimonios* – that which reflects conflict experienced among women in the movement who did not feel represented by the initiatives of many of the movement’s leaders. Vargas’ belief that “conflict is how a movement grows” is supported in evidence that over the past several decades the movement accelerated its focus on intersectionality and is now rooted in a firm understanding and respect toward difference. In sum, the women included in this section represent ways that conflict – stemming from varying sources (e.g., gender oppression, war, heterosexism) – has been viewed within the women’s movement as opportunities to take the movement, and it’s aims, further. Rather than conflict serving to justify the oppression and marginalization of women, Lourdes, Gahela, Tarcila, Tania, and Virginia all saw it as the impetus to form an organization, run for or serve in Congress, or promote activist scholarship that gained international recognition.

**Theme 2: Militancy: Taking an unapologetic women-centered feminist approach to social change.**

Interestingly, and unexpectedly, multiple feminist leaders in the sample identified themselves as militants, or as engaging in militancy, as a way to talk about their *feminist* action. This use of the terminology reflects a shift in strategies used to address gendered oppression. Whereby in the 1980s, militant labeling reflected identification with party politics, the current use appears to be a reclamation that draws attention to the fact that feminist activists are centering gender inequity in leftist politics. In this manner, women are using the term militancy to underscore an impassioned commitment, not as a literal reference to the use of militarized violence or authoritarian force. This is reflected by the testimony of a young former Congresswoman, Indira Huilca Flores, who focused her political work on policy and gender equity, when she shared that her most satisfying achievement was her militancy:

I can’t say what has given me more personal satisfaction because of course there are several measures. Someone could say that, well, I have been a congresswoman, I have had a responsibility as an authority and there are things that have been achieved from Parliament. I could tell you that, but I also think that there are things that cannot be compared in the same way. The very fact of having organized myself, of having been part of a space where I have militated in the left. A space that adopted the agenda that we feminists had been building, some things that perhaps 15 years ago would not have been so firmly enunciated from the left: the agenda in favor of sexual and reproductive rights, in favor of gender equality. Trying to also articulate that struggle that is not something isolated, but something that is really understood within a more comprehensive program of change within the left. That makes me very proud. The fact that this was incorporated to a certain extent in the organizational dynamics of the left seemed to me an achievement, it seems to me to be something important that I hope can be maintained.

Indira elaborates on this achievement in her testimonio by praising the establishment of women’s human rights on voters’ ballots and political discourses. She views the process of mobilizing for women’s human rights as a long-term iterative project, taking multiple decades to see the legislative impact of her militancy – or active commitment - within the movement. Moreover, as a woman in her 30s at the time of the interview, she demonstrates that identifying as a militant now does not require abandoning women’s causes, but rather includes unapologetic awareness and action surrounding gender inequity.

One characteristic of militancy, as it was described in the *testimonios*, was the extent to which identifying as ‘militant’ reflected the commitment leaders have for the women’s movement and women’s human rights, rather than reflecting a violent or armed forces approach. When women were self-identifying as a militant or invoking militancy, it was in the context of discussing a lifelong, sustained, and unapologetic commitment to working toward gender equity. For example, Tarcila Rivera (the CHIRAPAQ leader introduced earlier) expressed wide-ranging and extensive impacts of militancy on her life, altering even her way of being and viewing the world:

I was settling the whole process of my life. I had been drinking from the women’s and feminist movement because I participated from the time I started working in the State, participating in the unions. All that learning for me was foundational. At the age of 32, 33, when I returned from Italy, that is, since the 1980s, I was a militant, and then I dedicated myself first totally as a volunteer in the Indigenous movement to participating actively. I never wanted them to consider me as a leader, but to see the contributions I made because there were all male leaders…. So that’s when I started to have this conflict because my way of being, was not that of a submissive woman, waiting for men to tell her, this clashed with the way of leadership, which was totally macho. And then in ‘86, I began to look at it differently and we founded CHIRAPAQ. I always saw everything from an Indigenous perspective and I would say from a gender equity perspective…. The 2030 agenda^12^ is seen as an opportunity. In this ODS-5,^13^ how can we as Indigenous women insert ourselves or use this article so that our initiatives do not continue to be pilot experiences but rather become policies of the State. It is a very difficult job, it’s long, because it is important.

Tarcila recalls that dedication to the women’s and Indigenous movements, rather than submissiveness toward traditional leaders, was initially interpreted by male Indigenous leaders as a “violation” of traditional gender roles. She labels her commitment as “militant” to reflect a total dedication, even in the presence of “limit situations” that were characterized by machismo and conflict. Tarcila, along with others in the sample, identified themselves as militants to express a sustained pledge to mobilizing for gender equity, with long-term goals to address structural change. For Tarcila, this change would involve policy change at the State level that took seriously the efforts engaged by activists.

In sum, Indira and Tarcila both reflect a theme present in the interviews reflecting that within the contemporary women’s movement women see militancy as a way of *centering* women’s issues among leftist agendas, rather than marginalizing them to a feminist platform. Moreover, a militant perspective appears to be one that is not simply dedicated to change but sees the achievement of gender equity as one that can only be actualized if structural, or policy change, is enacted to give women guarantees. As such, many movement leaders utilized political platforms and ran for municipal, regional, and national political offices as part of their militancy.

**Theme 3: Pluralism: An approach that is inclusive of the diversity of women’s realities and experiences across generations and differences.**

Although others have written about the plurality present in the women’s movement in Perú in the form of “streams” (Vargas, 1991), the women in the current sample articulated nuanced ways in which their approaches and perspectives to plurality included recognizing the work of other feminists who came before them, the importance of solidarity across difference, and working with women networked in feminist organizations locally or internationally. Values rooted in pluralism were underscored in the interviews when women communicated that change cannot happen alone and that the differences of other women were paramount to launching a united cause of action against structural inequity.

Tarcila Rivera Zea, a Quecha activist, in her 60’s at the time of the interview, touched on each of these factors by noting who was of influence to her, centering her work in Indigenous communities, and articulating the importance of international solidarity:

I will always have a special affection for Ana Maria Portugal.^14^ She was the first feminist who thought that I should be there participating in different spaces. So I have been modestly and humbly learning and after Beijing,^15^ I began to look a little bit at what the women’s movement was, the feminist movement….

  …We had cultural affirmation workshops in the neighborhoods where the language was the vehicle for communication and the arts, right, of our elders with these children and also talking to the rural teachers about identity and food culture, combining the two. That has been a wonderful experience and from that project comes Tania Pariona.^16^ For me, she is the best example of our strategy. Since she was a child, she affirmed her identity, affirmed her cultural expressions, her leadership because she too has been a leader since she was a child, an adolescent, a young person and now an adult.

  .

  [our relationship with other women’s organizations has been one] Of mutual respect and dialogue. In other words, we are not exclusive. If we are invited, we go and we are, like Chirapaq, part of the Latin American women’s group that influences the Commission on the Status of Women.^17^

  …

  Then we have the Continental Link of Indigenous Women^18^ and I’m always pushing and after the Continental Link, we have the International Indigenous Women’s Forum^19^ which is global. So what I have done is try to carry out the learning and do it collectively and I think that Chirapaq has been an inspiration for many Indigenous associations that now exist. For example, I never called myself a leader or a director, no no I did not like to be told that I am a director because I don’t direct anything. I just carried out [laughter] my initiatives…. You have initiatives that you share, you carry them out, and in that sense, look at the international forum of Indigenous women, we are the geo-cultural regions of the world, we are, I don’t know, Africa, Asia, and we already have common priorities. It’s almost 40 years of life totally dedicated to it.

In her *testimonio*, Tarcila referenced mentor support as pivotal in her likelihood of participating in important international meetings (e.g., the United Nations meeting in Beijing). Moreover, working on parallel processes with women throughout the world was a prominent part of her *testimonio*. Her perspective on leadership reflects elements of liberatory processes when she denies credit as a leader, but rather describes a process of “initiation,” yet one that would not work if pluralism were not a vibrant strategy among actors whose common priorities were with shared initiatives.

Tania Pariona Tarqui, referenced in Tarcila’s testimonio above, is among the next generation of Quechua leaders, a social worker, politician, and human rights activist who was elected to Congress in 2016. In 2018 she was the President of the Women and Family Commission of the Congress. As an activist, she works extensively to establish social equality for Indigenous people, youth, and women. Tarcila, who was 36 at the time of the interview, described how strategies that were inclusive of others’ experiences were part of her foundational years:

Since a very young age, I have been incorporating myself into these organizational processes. …. In 2010, with Andean and Amazonian sisters, who were already engaged in a process of meeting and affirming Indigenous identity in Perú, we formed the *Organización Nacional de Mujeres Andinas y Amazónicas de Perú*, (ONAMIAP).^20^ I am the first youth secretary that ONAMIAP had. …. And at the international level, with the continental liaison of Indigenous women since 2004, we had already begun to participate in international spaces. For me, 2004 was a determining moment because it was the first time that I was able to meet Indigenous sisters from all over the world. From the American, Asian, and African continents, and for me that was a way to see myself reflected in my own history, in my own dreams, in my own life.

  .

  Since I was very young, I always had the idea that by being together we could have more strength. By being organized we could have a stronger voice, by being organized we can show that we can also request proposals. And my conviction to do things, not only thinking of myself but of others, is what I feel has led me to this….So, it has been several elements that have somehow shaped my personal commitment, always thinking about the collective.

  .

  For us, strengthening the organization has always been one of the priorities, because we knew that by having a strong organization, we could promote a next level of organization. We began as adolescents from peri-urban neighborhoods and then we formed a regional network that includes provinces which includes not only displaced-migrant youth but also youth who are in their places of origin and then articulate with Amazonian brothers, Aymaras at the level of Perú. So we have always considered that strengthening the organization has to be the first task, and perhaps as a strategy, that today becomes a **methodology** and institutional proposal of Chirapaq, which I am a part of.

  From that program, we accompany organizational processes from the community level to the most international level. …. And we have not lost the link with the organizational bases. On the contrary, I think that the work in the parliament allows me to continue being in contact with different sectors such as domestic workers, organizations of victims of violence from the 80’s to 2000, and migrant women in Lima.

Tania’s *testimonio* is a powerful illustration of how a younger generation of leaders benefited from and expanded upon the work that women who came before them produced. Because of the changes that women like Tarcila helped create, Tania could begin active processes of leadership from a young age. Tania explained that it was in youth that she developed a perspective rooted in plurality when she recognizes that together marginalized people have more strength. She explains that her values, and her strategy for change, are rooted in not only thinking about herself, but considering others – and the others include her elders, others she is organized with, as well as regional and international activists. Tania is explicit that a plurality of process is a strategy, or method, that helps strengthen the organization in which she works to create change. As a congresswoman, she pulled on these strategies and feminist acts of liberation to be an effective leader.

And, finally, Gahela Tseneg, introduced earlier, also expressed the importance of acknowledging the work contributed by earlier generations of active women and the value of working collectively across difference when she said this:

I don’t think I’ve ever worked alone. I come from a collective point of view and there have been several things we’ve done. And I say we because I think that the things that we can do have to do with what women have started throughout history, right? So what Micaela Bastidas^21^ has done is what Adela Montesinos,^22^ María Jesús Alvarado,^23^ Zoila Aurora de Cáceres^24^ and many others have continued to do. It has helped us to talk about these issues today, so that we can participate in social organizations, so that we can participate in political parties, so that we can vote, so that we can demand, so that we can even have a voice to speak out and make demands. I have participated in a congressional campaign; the fact that I have been so close is not an individual achievement, it has to do with an achievement of women like María Elena Moyano,^25^ it has to do with the work that Rosa Mavila,^26^ Verónika Mendoza^27^ has done, with which Indira Huilca,^28^ Marisa Glave^29^ has continued in the last congress; Tania Pariona^30^ women who continue that. We can have discrepancies and differences, we can not agree on everything but I think there is a key structural factor that unites us: breaking this murderous system. I believe that the wave is bigger and bigger, the snowball is growing, and at some point, we will be so many that they will not be able to contain or silence us.

Gahela recognizes that challenging social structures related to inequity involves confronting the social situation that disenfranchises women across discrepancies and difference. A prominent element of the *testimonios* in this sample, and which was reflected in Tarcila, Tania, and Gahela’s words, was the regularity with which women pointedly credited other women’s work. Many women paid homage to women from prior generations who were pioneers of feminism, many made clear that their activism was deeply interconnected with the work of others, and others still gave specific acknowledgment to women in the movement who were influential or key actors in their own growth as actors. None of them spoke of doing it “alone.”

## Discussion

The standpoints represented among the feminist activists in this study underscored how diverse histories and experiences were important to the demand of social, organizational, and governmental transformations related to gender equity. Through the use of *testimonio*, we learned how perspectives and experiences from various backgrounds shaped women’s activism and contributed to the redistribution of political power that has been central to processes involving equity in Perú. The findings revealed three key components of feminist activism as articulated by the social movement actors themselves: conflict, militant identity, and pluralism. In analyzing why these components held importance for the potential for change, there were several processes identified.

First, it was found that acknowledging and resisting *conflict* was an important part of the process for women attempting to gain access to and control over an equitable share of decision-making. The feminist activists interviewed in Perú demonstrated that awareness of and dialogue about conflict situations led to action in a cyclical manner that addressed the social conditions women confronted associated with equity. Some of those conditions involved being marginalized from community decision-making, some of them involved discrimination, others involved political violence, and others still involved tensions in Indigenous communities. In developing the capacity to resist oppression and engage in decision-making, the women in this sample pursued leadership as congresswomen, scholars, and leaders of organizations. If the women had not viewed conflict as an opportunity to exert their right to participate, they would not have emerged as leaders contributing to an equitable society. In other words, they took power that was not given, not even by a progressive left, rather than viewing conflict as a closed door. That this first theme was lengthier reflects the primacy of this experience and that it was discussed by a majority of women in the sample.

Second, the findings regarding *militancy* suggest an identity departure away from leftist, militant political affiliation, instead of articulating a militancy that imparts a responsibility to engage in collective action for gender equity, specifically. The militant identity, articulated in this sample, in contrast to how it was used in the 1980s, reflected an identity that unapologetically centered women’s issues in the political agenda. Perhaps more importantly, and what others the world over can learn from, is that feminist activists who identified themselves as militants were not invoking a combative stance, but rather one that reflected a sustained pledge to address gender equity through structural change. In other words, a militant perspective would not be satisfied with simply mobilizing or raising awareness of women’s experiences, but would instead work toward gender equity being reflected in “policies of the State,” recognizing that structural transformation was necessary for a “comprehensive program of change within the left.”

The third component in the processes that were identified by women in this sample was that activists resisted power-based constraints by taking an approach that valued a *plurality* of experiences and social locations from which to work. Specifically, in detailing a plurality of process, women in Perú demonstrated how the solidarity they established across difference, sectors, regions, and countries was a critical piece to sustaining efforts, gaining legitimacy, elevating their voices, and creating policy change. The feminist processes that were shared detailed numerous ways that women identified how change efforts resulted through a deep connection to each other’s work and solidarity. The plurality of process was a strategy, not an accident.

In addition to broadening feminist theory through the inclusion of voices, it is perhaps more important that these processes have global implications for change. Including majority world women’s perspective in the construction of feminist knowledge not only serves to refute a “victim” narrative that women need outside help to rectify injustice, but it in fact opens the possibility for understanding how a majority world perspective, inexplicably linked to systematic inequities of global power (e.g., colonialism, globalization; Lugones, 2010; Sen and Grown, 2013), can develop important strategies for change that could inform efforts the world over. Throughout the world, limited opportunities for women’s political participation and decision-making reflect a widespread societal problem. Despite decades of international urgency directed toward the enhancement of women’s political participation, the global average of women in representative governmental positions increased from 9% in 1987 to only 25% in 2020 (Cornwall and Goetz, 2005; World Bank, 2021). Though the number nearly tripled over the course of three decades, the current percentages still reflect staggering underrepresentation of women. Scholars have suggested these numbers remain low because the political landscape for women, throughout the world, offers predominately nominal support for their involvement and often does so without considering the gendered inequities and discrimination that women confront (Mohanty, 2007). *Testimonio* from the current study suggests that interrupting structural inequities that are institutionalized, in solidarity with others, is a critical step toward getting women in decision-making positions (i.e., Congress) where they can use their power or authority to either raise awareness or legislation surrounding gender inequity. The legislation that was put forward by feminist activists in Perú (and that was mentioned in the testimonios), the National Gender Equality Policy and the Gender Parity Law, recognize the institutional nature of gender discrimination. This is an important finding to consider in the context of what is happening elsewhere in the world. For example, in the United States, a Presidential Executive Order issued in 2020 attempted to silence conversations about gender equity by prohibiting the use of “divisive” concepts, such as sexism (or racism), and denounced or restricted funding to federal workforce employees who promoted views related to gender inequity. Relatedly, at the time of this writing, eight of the United States have passed bills based off of the Executive Order that ban the teaching of racial inequity, with 15 additional states considering similar legislation (Ray and Gibbons, 2021). Findings from the current study suggest that progress lies in *challenging* the institutionalization of inequity, rather than upholding the *status quo* through legislation. The activists in Perú demonstrated that having women confront conflict, maintain a militant identity in the face of it, and operate through a plurality of processes were successful means to confronting structures of inequity and taking steps to pass protective legislation.

Finally, findings from the current study have implications for women’s political participation in global governance as well, such as within the United Nations. For example, in international governance contexts, such as peacekeeping, women’s participation falls short of parity both in terms of female soldiers and in the mainstreaming of gender experts (Reeves, 2012). Although the United Nations recognizes that sustainable peace is “inextricably linked to equality between women and men” (United Nations, 2004), gender experts for the United Nations report that feminist knowledge related to gender equity is sidelined to the greater interest of ending conflict. As such, “talking like a feminist” closes dialogue and results in the marginalization of feminist knowledge. Therefore, numerous examples the world over exist whereby women continue to be marginalized in areas of political decision-making.

## Limitations and Conclusion

Although the use of *testimonio* allows for invisible and often undocumented lived experience to contribute to the construction of knowledge, the method is nevertheless limited by small sample size and generalizability. Findings would need to be interpreted for application in local contexts. Another limitation is that although the study was designed to center majority-world women’s voices, the analysis and conclusions were filtered through the author’s social location as a white, middle-class woman from the United States. The limited contextual embeddedness and “outsider” status of the author informed the use of *testimonio* and reliance on liberation psychology as a means for understanding how women resist power-based constraints. The positionality of the author also had the potential to impact the interpretation of the participants’ accounts; for example, the three themes identified are all principles that are ripe for growth in the minority world, in which the author is situated.

In sum, the findings from activists in Perú have the potential to provide a framework for resisting marginalization by demonstrating that challenging conflict, adopting an unapologetic stance to ending systemic gender-based power imbalances, and working in solidarity across difference was critical for women’s political participation. Specifically, these processes led to women gaining seats at the table (i.e., as organizational leaders, as politicians), and occupying those seats was critical to influence practice, policy, and national legislation. The findings from the current study offer keys to success for pervasive problems rooted in gender inequity that continue to persist around the world.

## Data Availability Statement

The raw data supporting the conclusions of this article will be made available by the authors, without undue reservation.

## Ethics Statement

The studies involving human participants were reviewed and approved by the Institutional Review Board UCSC. The patients/participants provided their oral informed consent to participate in this study.

## Author Contributions

The author confirms being the sole contributor of this work and has approved it for publication.

## Conflict of Interest

The author declares that the research was conducted in the absence of any commercial or financial relationships that could be construed as a potential conflict of interest.

## Publisher’s Note

All claims expressed in this article are solely those of the authors and do not necessarily represent those of their affiliated organizations, or those of the publisher, the editors and the reviewers. Any product that may be evaluated in this article, or claim that may be made by its manufacturer, is not guaranteed or endorsed by the publisher.

## Footnotes

1 Given that the terms “developing” and “third world” are often used by so called “first world” nations to describe another country in a manner that implies inferiority, the term majority world, borrowed from Kagitcibasi (2002) will be used because individuals from “developing” countries constitute the majority of the world’s population. Moreover, terms like Global North/South, which attempting to address this issue by using geographical references, are simply not accurate as proxies for economic development because many rich countries exist in the South. Therefore, “majority world” is both less pejorative and more accurate.

2 One of the directors of the Flora Tristán center was the community collaborator for the current study.

3 Brazil, China, Germany, India, Nicaragua, Nigeria, Poland, Russia, and the United States. https://sites.lsa.umich.edu/globalfeminisms/

4 The undergraduate research assistants were part of a University of California initiative, the Building Belonging program. The program was a 5-year effort designed to increase engagement and build a greater sense of belonging for under-represented undergraduate students through faculty mentored service-learning and research projects. The program was offered through the Institute for Social Transformation in the Division of Social Sciences. Credit to the students involved in transcribing the testimonios goes to: Jessica Valdez Alvarez, Karely Valdez Lopez, Lizbeth Garcia, Maria Govea Mendoza, and Nicole Herrera-Moro.

5 The National Federation of Female Peasants, Artisans, Indigenous, Native and Salaried Workers of Perú, FENMUCARINAP, was founded in 2006 with the purpose of defending and fighting for the rights of women in Perú.

6 Diana Miloslavich is a director at Flora Tristán. She was the community collaborator for this study.

7 Estuido para la Denfense de los Derechos de la Mujer (DEMUS: Study for the Defense of Women’s Rights) is a Peruvian feminist organization that defends human rights, in particular the sexual and reproductive rights of women, promoting their free exercise and questioning of the hegemonic cultural paradigm on women and their sexuality.

8 Confederación Campesina del Perú (CCP) is a farmer’s movement that was founded in 1947.

9 Activists in the women’s movement had been working on the gender parity law for political participation. In August 2020, President Vizcarra signed a law establishing gender parity in congressional elections. The law requires that 40% of congressional candidates be female by 2021, 45% by 2026, and 50% by 2031.

10 The Shining Path began as a political party in Perú during the 1980s. It grew into a militarized organization that used guerilla warfare, brutality, and civilian massacre to terrorize and gain control over areas of Perú. The Shining Path seized control over large portions of Perú through force for a decade.

11 A non-governmental organization (NGO) operates independently of the government and often has a stated purpose to address a social or political issue.

12 The United Nations set a goal for sustainable development by 2030 meant to transform the world. The agenda for 2030 is a plan of action for people, the planet and for prosperity. It seeks to strengthen universal peace.

13 Objectivo de Desarrollo Sostenible 5 (ODS-5); Sustainable Development Goal 5 (SDG-5) is one of the 17 Sustainable Development Goals (SDGs) established by the United Nations in 2015. This goal focuses on empowering women and girls in order to reduce gender inequality.

14 Ana Maria Portugal is a native of Arequipa, a writer and journalist, a pioneer of feminism in Perú and Latin America. She is the co-founder of the Centro de la Mujer Peruana.

15 The 1995 Fourth World Conference on Women in Beijing, China marked a significant turning point for the global agenda for gender equality. More than 30,000 activists and representatives of 189 countries gathered to debate what it would take to make a gender-equal world. Together they created the Beijing Declaration and Platform for Action, the most comprehensive agenda to date on gender equality and women’s empowerment. Many feminist activists in Latin America either attended the Beijing conference, or use it as reference point.

16 Tania Edith Pariona Tarqui, referred to earlier in the manuscript as a participant in this study, is an example of a Quechua leader and activist for human rights who learned from the prior generation.

17 The Commission on the Status of Women of the United Nations is the principal international intergovernmental body devoted exclusively to the promotion of gender equality and the empowerment of women.

18 The Continental Network of Indigenous Women of the Americas, ECMIA, is a network of women’s and Indigenous peoples’ organizations that have been promoting the recognition and respect for the rights of women, youth, children, and Indigenous peoples throughout the Americas for 20 years.

19 The International Indigenous Women’s Forum is a global network that brings together Indigenous women’s organizations, leaders and human rights activists from Asia, Africa, the Pacific, the Arctic, and the Americas to agree on agendas, strengthen capacities and leadership in international decision-making processes.

20 Organization that promotes the participation of Indigenous women and the fulfillment of their individual and collective rights. http://onamiap.org/

21 Micaela Bastidas Puyucahua (1744–1781) was an Indigenous rebel leader, fighting against Spanish colonial rule alongside her husband Tupac Amaru II. She helped to involve hundreds of Indigenous women in the struggle against the oppression of the Spanish mandate.

22 Adela Montesinos (1910–1976) published in newspapers at the age of 19 by using a pseudonym, participated in the founding of a Socialist Party in 1929, and in 1931 became the first woman to speak at a political party demonstration.

23 María Jesús Alvarado Rivera (1878–1971) was regarded as a rebel feminist, educator, journalist, writer and social activist. She was noted by the National Council of Women of Perú in 1969 as the “first modern champion of women’s rights in Perú.”

24 The writer Zoila Aurora Cáceres (1877–1958) was one of the first and most prominent feminists of Perú in the first half of the 20th century. Her commitment to the defense of women’s rights had a greater impact on Peruvian society than on her literary work.

25 María Elena Moyano Delgado (1958–1992) was an Afro-Peruvian community organizer and feminist who was assassinated by the Shining Path. She served as president of a women’s organization twice and was a deputy mayor at the time she was murdered. She received the Peruvian Order of Merit posthumously.

26 Rosa Mavila (1952-) was a congresswoman from 2011 to 2016.

27 Veronika Fanny Mendoza Frisch (1980-) is a French-Peruvian psychologist, anthropologist and politician. Since December 2017 she has been president of the New Perú movement. She was a candidate in the 2016 presidential election.

28 Indira Isabel Huilca Flores (1988-) is a Peruvian socialist sociologist and politician, feminist and progressive activist. She was a congresswoman from 2016 to 2021. In the 2016–2017 legislature, she was President of the Women and Family Commission. In the 2018–2019 period, she served as head of the Women and Family committees; Labor and Social Security and as Secretary of the Justice and Human Rights Commission. Indira was a participant in the current study.

29 Marisa Glave Remy (1981-) is a Peruvian sociologist and politician. She served in the Peruvian Congress from 2016 to 2019. Previously, she was a city councilor in the city of Lima, and was a founding member of the Earth and Freedom movement, currently called Frente Amplio por la Justicia, la Libertad y la Vida (Broad Front for Justice, Freedom and Life).

30 Tania Pariona Tarqui (1984-) is a Quechua leader, social worker, politician and human rights activist who was elected to represent the Department of Ayacucho in the Congress of the Republic of Peru in 2016. In 2018, she was the President of the Women and Family Commission of the Congress. As an activist, she works extensively to establish social equality for Indigenous people, youth, and women. Tania was a participant in the current study.
